# Comparative Study of Heavy Metals in Dried and Fluid Milk in Peshawar by Atomic Absorption Spectrophotometry

**DOI:** 10.1155/2014/715845

**Published:** 2014-05-20

**Authors:** Ghosia Lutfullah, Abid Ali Khan, Azra Yasmeen Amjad, Sajida Perveen

**Affiliations:** ^1^Centre of Biotechnology & Microbiology, University of Peshawar, Peshawar 25120, Pakistan; ^2^Department of Chemistry, COMSATS Institute of Information Technology, Abbottabad 22060, Pakistan; ^3^Department of Nutrition, College of Home Economics, University of Peshawar, Peshawar 25120, Pakistan; ^4^Department of Environmental & Soil Sciences, Agricultural University, Peshawar 25120, Pakistan

## Abstract

Various essential and toxic heavy metals (Ca, Mg, Cu, Zn, Fe, Mn, Pb, Cd, Cr, and Ni) contents in various types of dried (*infant formula* and *powdered*) and fluid (*fresh* and *processed*) cow milk were assessed by atomic absorption spectrophotometry. The milk samples were collected from local markets of different parts of Peshawar city, Pakistan. Heavy metal concentrations varied significantly depending upon the type of milk. The heavy metal concentrations in most of the samples were within normal and permissible ranges. It was observed that the samples contained considerable amounts of calcium, while magnesium levels were well above the required levels. The results also revealed that copper levels were slightly lower than the permissible limits. The concentration of zinc in dried milk samples was greater than the values for the liquid milk types. Infant milk formulae had higher iron levels as compared to other milk samples because of the added constituents. Significant differences were observed in the mean values of manganese and cadmium in different types of milk. The toxic metals were within the acceptable limits and did not show significant levels leading to toxicity.

## 1. Introduction

Milk is the first food that human encounters which serves as a source of essential nutrients required for the biological functions and growth during early stages of life [1], so commercial infant formulae are designed to mimic the composition of human milk [2]. Formula requiring dilution needs to be measured to correct proportions as the dangers of imbalance exist if excessively concentrated formula or undiluted cow's milk is fed because of preparation error or ignorance [3]. Evaporated and commercially prepared formulae are more expensive than fresh or liquid milk [4]. The infant formulae sometimes lead to nutrient imbalance, poor bioavailability, and hypersensitivity [5]. The bioactive peptides in milk minimize the risk of chronic diseases by boosting immune system [6].

In formula milk, essential elements are added to satisfy nutritional requirements; however, too high additions of certain elements are detrimental, and milk may serve as a vector for the transmission of toxic substances of extrinsic origin [7]. The residues of various antibiotics and antimicrobial drugs may enter milk through medicated feed and improper use of intramuscular drugs. “*Essential metals*” (Ca, Fe, Zn, Cu, and Se) are those required for the complete life cycle of an organism, and absence or insufficiency of which in human diet could induce modifications of metabolic changes and some diseases. However, some essential metals become “*toxic*” when their concentration is increased, especially the levels exceeding by 40-to-200-fold [8]. If their intakes via food chain cross the permissible levels [9], toxicity may be a problem. The severity of toxic effect depends on nature and concentration of the elements, body resistance, and antagonistic effects of other chemical contaminants [10]. The occurrence of elements in foods is a function of the biological roles played by the elements in the structure and physiology of the food issue and adventitious contamination during growth, processing, and preparation. Commercial dried milk and infant formula are deliberately fortified with essential elements such as iron, zinc, and copper to ensure proper nutrients provision [11]. The assessment of heavy metal exposure in the infants' diets is very essential so as to have a clear picture of the intakes, utilization, and retention of essential as well as toxic nutrients.

Multielement surveys have been carried out on trace element concentrations in infant foods, human milk, and powdered infant formula determining mineral elements in the digestion solutions [12]. Concentrations in animals may increase due to environmental contamination [13], resulting in toxic elements in foodstuff [14]. Infants and young children may absorb 50% of dietary toxic metals as compared with only 10% by adults [15]. Heavy metals are dangerous because they tend to bioaccumulate in a biological organism faster than being metabolized or excreted [16, 17]. Some metals form stable covalent complexes and interact with macromolecules with affinity for organic binding [18], possessing damaging action at the molecular level. Regulations on milk quality depend upon accurate and precise milk composition [19]. Metals affect the intensity of the Maillard browning reaction in milk. Fe^++^ ions stimulate while Cu and Zn suppress browning of the milk mixtures [20, 21].

The objectives of the present study were to determine the levels of various essential and toxic trace elements in cow milk from local markets of Peshawar and also to compare their concentration in infant formula/dried milk with fresh/processed liquid cow milk.

## 2. Materials and Methods

A total of forty-six samples from dried, infant formula, fresh, and liquid milk were randomly selected and analyzed in year 2009 for determination of essential and toxic (Ca, Mg, Cu, Zn, Fe, Mn, Pb, Cd, Cr, and Ni) heavy metals. The samples were procured from the local markets of Peshawar. Individual samples of 50 gm of dried and 100 mL of liquid cow milk were selected in sterile plastic containers and then were labeled, sealed, and stored at suitable cold temperature (0–5°C). The sample treatment involved digestion, extraction, and preparation of samples before atomic absorption analysis. The HNO_3_/HClO_4_ based wet digestion process was adopted for the identification of trace elements by atomic absorption spectrophotometry as described by AOAC (2000). The temperature-time combinations were optimized for each element, and the accuracy, precision, selectivity, and sensitivity were verified by using* Perkin Elmer Double Beam 2380 Atomic Absorption Spectrophotometer* with reference sample. The blanks were made in the same way without using any sample. The filtrate was stored in properly labeled and sealed plastic bottles. All the samples were prepared in triplicate.

## 3. Statistical Analysis

The data was analyzed by using Excel data sheets and SPSS computer software version 17. The simple percentages plus mean values + SD of the heavy metals were calculated. One-way analysis of variance (ANOVA/LSD) was applied to find out statistical differences among various parameters in different milk samples. The data was also analyzed in terms of Pearson's logarithmic correlation (<0.05 and <0.01) to indicate the strength level of the correlation among various heavy metals.

## 4. Results 

Milk is a valuable source of essential minerals but some toxic elements may be accidently added during handling, processing, and remixing of milk. The mean concentrations of various essential and toxic heavy metals are presented in Tables 1 and 2 indicating mean values + SD and differences (ANOVA/LSD) among the various categories of milk. Mean values of the mineral elements were closest to the permissible values as determined by international standards [22–24]. The differences in mean values (Ca, Mg, Cu, Zn, and Fe) of the four types of milk were observed to be nonsignificant, whereas there were significant (*P* < 0.05 ANOVA/LSD) differences in case of manganese. The mean differences of cadmium were significant in the dried and liquid milk groups, while nonsignificant differences among mean values of lead, chromium, and nickel of the types of milk were observed. The results of the correlation coefficient as presented in Table 3 show the correlations of some minerals with others at different levels of significance.

## 5. Discussion

The concentrations of calcium in all types of milk were of the same order of magnitude as reported in the literature [23], and there were no significant differences in mean values between the two types of each milk group: the powdered milk and the liquid milk. Magnesium being a major mineral required for the regulation of different body processes [10] was found in accordance with the given values [24]. Copper being considered a very important cofactor in the effective utilization of several elements especially iron in body [11] was observed to be slightly lesser than the standards. Zinc as an important constituent in promoting growth and regulation [25] was sufficiently found in most of the samples. Though milk is the poorest source of iron, it performs very important functions in the body, and the calcium in milk itself favors the absorption of dietary iron. It has been observed [26] that low iron status imposes cadmium burden on body, so special attention is required to ensure adequate iron intake to reduce cadmium absorption. The imbalance of iron has been observed to decrease immune functions and to release browning compounds and the oxidized form of sulfur containing amino acids; that is why infant milk formula and some powdered milk samples were found to have added constituents [6].

Toxic metals are enzyme toxins that disturb the immune mechanism [18], so they need to be eliminated through treatment which can restore the immune system to the maximum effective level. Lead is among the main metals present in the environment which have major toxic effects. Its increased levels have been associated with learning deficiencies in children [2]. There were nonsignificant differences between milk samples. Generally, infant formula reconstituted with tap water is at the highest risk from metal-containing water supply [24]. Deficiency of calcium, iron, and zinc enhances the effects of lead on cognitive and behavioral development. Iron deficiency increases cadmium absorption that affects zinc utilization [27]. Toxic effects of metals may be mediated or enhanced by interaction or deficiency of nutritionally essential metals [20].

The correlation coefficient analysis (Table 3) further verified that calcium was significantly correlated (<0.05) with magnesium. The correlation coefficient analysis explained that copper was significantly correlated (<0.05) with magnesium and calcium. Manganese was only found to be significantly (<0.05) correlated with cadmium. The correlation analysis summarized the data showing lead as highly correlated (<0.01) with iron and chromium at <0.05 significance level. Cadmium was found to be nonsignificantly (<0.05) correlated with other metals. The correlation coefficients stated that chromium was nonsignificantly correlated with all the metals (<0.05). Nickel was found to be only correlated (<0.05) with zinc and chromium.

## 6. Conclusion

The results of the study provide valuable data regarding essential and toxic heavy metals in various powdered and liquid milk samples available in the market. It was observed that the determined values were within the acceptable limits with significant differences in mean values of manganese and cadmium in different types of milk. The milk samples contained considerable amounts of calcium, while magnesium levels were well above the required limits. Copper levels were slightly lower than the permissible allowances. The concentrations of zinc in dried milk samples were up to the mark and better than the liquid types. Infant milk formulae had better iron levels as compared to other milk samples. The correlation coefficients showed that most of the metals were significantly correlated (>0.05) with each other.

## Figures and Tables

**Table 1:** Concentration of essential mineral elements (ppm) in milk.

Milk group		Essential minerals					
		Calcium	Magnesium	Copper	Zinc	Iron	Manganese
	**Total**						
	Mean + SD	2106 + 954	349 + 205	0.59 + 0.59	4.79 + 4.03	1.67 + 2.08	0.0267 + 0.02
	Range	970–3825	110–875	0.10–2.37	0.09–12.80	0.02–7.54	0.01–0.09

Dried	**Infant formula**						
	Mean + SD	3081 + 277 NS	540 + 206 NS	1.00 + 0.76 NS	8.97 + 2.35 NS	4.33 + 2.22 NS	0.0318 + 0.02*
	Range	2475–3600	250–825	0.11–2.37	4.69–11.34	1.33–7.54	0.01–0.07
	**Powdered**						
	Mean + SD	3092 + 502 NS	480 + 173 NS	0.90 + 0.41 NS	7.31 + 4.20 NS	2.42 + 0.99 NS	0.0400 + 0.02*
	Range	2075–3825	225–875	0.10–1.40	2.22–12.80	1.30–4.30	0.02–0.09

Liquid	**Fresh**						
	Mean + SD	1333 + 142 NS	240 + 106 NS	0.15 + 0.01 NS	1.93 + 1.45 NS	0.17 + 0.12 NS	0.0215 + 0.01*
	Range	1050–1580	120–425	0.12–0.17	0.69–5.01	0.02–0.43	0.01–0.04
	**Processed**						
	Mean + SD	1228 + 204 NS	185 + 50 NS	0.46 + 0.52 NS	1.97 + 1.73 NS	0.25 + 0.11 NS	0.0166 + 0.01*
	Range	970–1725	110–250	0.11–1.70	0.09–4.84	0.06–0.43	0.01–0.03

*Significant at *P* < 0.05 (ANOVA/LSD).

NS: nonsignificant.

**Table 2:** Concentration of toxic mineral elements (ppm) in milk.

Milk group		Toxic mineral elements			
		Lead	Cadmium	Chromium	Nickel
	**Total**				
	Mean + SD	0.0091 + 0.01	0.1507 + 0.28	0.0007 + 0.00	0.0146 + 0.01
	Range	0.0001–0.03	0.01–1.20	0.0001–0.003	0.002–0.032

Dried	**Infant formula**				
	Mean + SD	0.0177 + 0.01 NS	0.3545 + 0.40*	0.0015 + 0.00 NS	0.0277 + 0.00 NS
	Range	0.01–0.03	0.09–1.18	0.001–0.003	0.022–0.032
	**Powdered**				
	Mean + SD	0.0170 + 0.01 NS	0.2110 + 0.35*	0.0014 + 0.00 NS	0.0202 + 0.01 NS
	Range	0.01–0.03	0.04–1.20	0.001–0.002	0.002–0.032

Liquid	**Fresh**				
	Mean + SD	0.0035 + 0.01 NS	0.0408 + 0.07*	0.0001 + 0.00 NS	0.0065 + 0.00 NS
	Range	0.0001–0.015	0.01–0.25	0.0001–0.0003	0.003–0.009
	**Processed**				
	Mean + SD	0.0006 + 0.00 NS	0.0325 + 0.02*	0.0001 + 0.00 NS	0.0066 + 0.00 NS
	Range	0.0001–0.0015	0.01–0.09	0.0001–0.0003	0.004–0.009

*Significant at *P* < 0.05 (ANOVA/LSD).

NS: nonsignificant.

**Table 3:** Correlation coefficients of the mineral elements.

		Ca	Mg	Cu	Zn	Fe	Mn	Pb	Cd	Cr	Ni
Ca	Pearson's correlation	1	0.684**	0.502**	0.732**	0.729**	0.466**	0.767**	0.373*	0.771**	0.798**
	Sig. (2-tailed)		0.000	0.000	0.000	0.000	0.001	0.000	0.011	0.000	0.000

Mg	Pearson's correlation	0.684**	1	0.214	0.693**	0.485**	0.266	0.657**	0.173	0.722**	0.550**
	Sig. (2-tailed)	0.000		0.153	0.000	0.001	0.074	0.000	0.250	0.000	0.000

Cu	Pearson's correlation	0.502**	0.214	1	0.423**	0.521**	0.547**	0.469**	0.194	0.512**	0.460**
	Sig. (2-tailed)	0.000	0.153		0.003	0.000	0.000	0.001	0.195	0.000	0.001

Zn	Pearson's correlation	0.732**	0.693**	0.423**	1	0.639**	0.267	0.670**	0.460**	0.821**	0.570**
	Sig. (2-tailed)	0.000	0.000	0.003		0.000	0.073	0.000	0.001	0.000	0.000

Fe	Pearson's correlation	0.729**	0.485**	0.521**	0.639**	1	0.303*	0.480**	0.492**	0.681**	0.730**
	Sig. (2-tailed)	0.000	0.001	0.000	0.000		0.041	0.001	0.001	0.000	0.000

Mn	Pearson's correlation	0.466**	0.266	0.547**	0.267	0.303*	1	0.516**	0.064	0.326*	0.378**
	Sig. (2-tailed)	0.001	0.074	0.000	0.073	0.041		0.000	0.672	0.027	0.010

Pb	Pearson's correlation	0.767**	0.657**	0.469**	0.670**	0.480**	0.516**	1	0.257	0.729**	0.735**
	Sig. (2-tailed)	0.000	0.000	0.001	0.000	0.001	0.000		0.085	0.000	0.000

Cd	Pearson's correlation	0.373*	0.173	0.194	0.460**	0.492**	0.064	0.257	1	0.488**	0.433**
	Sig. (2-tailed)	0.011	0.250	0.195	0.001	0.001	0.672	0.085		0.001	0.003

Cr	Pearson's correlation	0.771**	0.722**	0.512**	0.821**	0.681**	0.326*	0.729**	0.488**	1	0.746**
	Sig. (2-tailed)	0.000	0.000	0.000	0.000	0.000	0.027	0.000	0.001		0.000

Ni	Pearson's correlation	0.798**	0.550**	0.460**	0.570**	0.730**	0.378**	0.735**	0.433**	0.746**	1
	Sig. (2-tailed)	0.000	0.000	0.001	0.000	0.000	0.010	0.000	0.003	0.000	

**Correlation significant at the 0.01 level.

*Correlation significant at the 0.05 level.
